# Mobile care app development process: using the ADDIE model to manage symptoms after breast cancer surgery (step 1)

**DOI:** 10.1007/s12672-023-00676-5

**Published:** 2023-05-09

**Authors:** Aydanur Aydin, Ayla Gürsoy, Hasan Karal

**Affiliations:** 1 Faculty of Health Sciences, Nursing Department, Gumushane University, Gumushane, Turkey; 2 Faculty of Health Sciences, Nursing Department, Antalya Bilim University, Antalya, Turkey; 3 Faculty of Education, Computer and Instructional Technologies Education, Trabzon University, Trabzon, Turkey

**Keywords:** Mobile app, ADDIE, Self-care, Breast cancer, Breast cancer surgery, Breast care nurse

## Abstract

The use of mobile applications is widespread in patient monitoring or education today. This study aims to describe the design and development process of a mobile app that supports patient self-care after breast cancer surgery. We used the ADDIE model to develop and test the mobile app. ADDIE (Analysis, Design, Development, Implementation, Evaluation) is a systematic approach based on a standard instructional design model for creating training materials. The model consists of five phases, each with its own set of steps. Once the steps within each phase are completed, the model progresses to the next phase, ultimately resulting in a “usable” product. Different team collaborations were established within each phase, and support was obtained from multiple experts during the design process. Thanks to this model, the information that patients need was transformed into a technological product. This article, which explains the stages of the product design process for mobile applications, provides information that may be helpful to researchers working on similar products.

## Introduction

The use of technology in healthcare has advanced significantly, similar to other fields [1]. These advancements offer various options for healthcare providers in diagnosing, treating, and monitoring patients [2]. Among the most effective ways to bring these options to users is through smartphones. Smartphones are portable, multi-purpose devices that incorporate technology into our daily lives seamlessly. They serve as computers, cameras, video and voice recorders, music players, radios, and navigation devices, in addition to communication tools [3]. Mobile health (mHealth) has the potential to transform healthcare by improving access to care, enhancing patient engagement and self-management, facilitating communication between patients and providers, and enabling remote monitoring and telemedicine. However, mHealth also presents challenges related to privacy, security, regulatory compliance, and equitable access. Just like any healthcare intervention, careful consideration of privacy and security measures, as well as compliance with applicable regulations, is crucial to ensure the safe and effective use of mHealth technologies [4, 5].

In recent years, people have increasingly relied on the internet and mobile apps for accessing information, leading to a decline in the use of printed media such as brochures and books. [6]. Advancements in technology have made it possible to easily install various medical mobile applications (apps) containing health information on smartphones [7]. Studies have shown that the use of mobile apps in healthcare has become widespread among the public, with easy access to these apps contributing to improved healthcare management [8, 9]. According to another study, the use of mobile apps in healthcare by the public has reached an acceptable level [10]. Many individuals use these apps for various purposes, such as receiving daily diet and fitness suggestions, tracking their steps, and setting reminders to drink water. These are being integrated into patient care, health management, and communication among healthcare professionals [11, 12]. Based on this information, it is anticipated that mobile apps can assist patients in managing problems that may arise outside of the hospital, ensuring their active participation in treatment, as well as facilitating consultations and communication.

Mobile apps are utilized for managing health problems in various disease groups [5, 13]. For instance, there are apps developed for skin cancer examination, enabling early detection and avoidance of potential side effects during therapy [14]. Similarly, mobile apps designed to monitor blood glucose levels in diabetic patients have been found to help maintain glycemic control and provide valuable data to clinics for timely intervention [15]. Mobile apps have been observed to be used in various categories including mental health, rare diseases, disease tracking, women's health, and infectious diseases [16, 17]. It is important to ensure that the utilization of mobile applications for disease management is grounded in evidence-based practices and aligns with applicable regulations and guidelines. However, it should be noted that not all healthcare apps are regulated, and patients and healthcare professionals should exercise caution to ensure they choose reputable and secure apps that comply with relevant healthcare regulations and standards [18, 19].

There are many mobile applications related to breast cancer that provide features such as informing patients, tracking their progress, and including exercise programs [20]. In a study, a mobile app was found to be effective in supporting breast cancer patients in continuing shoulder and arm exercises following axillary dissection [21]. Another study conducted with breast cancer patients revealed that a mobile app developed to report symptoms and collaborate with the hospital had a positive impact on patients’ acceptance of the disease and their treatment process [22]. There are limited mobile applications that provide education on self-care for breast cancer patients. It is worth noting that in our country, verbal training is commonly preferred in patient education, although other methods such as pamphlets, individual counseling, and brochures are also used [23, 24]. Also, we did not reach any Turkish app developed by a healthcare professional for breast cancer treatment [20]. For this reason, there is a need to explore mobile apps' effectiveness in providing support to breast cancer surgery patients.

This research aims to explain the development and evaluation of a mobile app to support self-care in patients undergoing breast cancer surgery. The research issue involves investigating whether a mobile app, based on the standard educational design method (ADDIE), can effectively provide information and support to patients and contribute to positive outcomes in terms of self-care behaviors.

Objectives:

- To conduct a thorough analysis of the educational needs and preferences of breast cancer surgery patients to inform the design and content of the mobile app.
- To design the mobile app using the ADDIE (Analysis, Design, Development, Implementation, Evaluation) instructional design model, ensuring it follows best practices for educational design.
- To collaborate with healthcare professionals, including surgeons, oncologists, and nurses, to review and validate the content and accuracy of the information provided in the mobile app.
- To develop a user-friendly and visually appealing mobile app interface that is accessible on both iOS and Android platforms, taking into consideration the diverse needs of breast cancer surgery patients, including age, literacy level, and technological proficiency.
- To create interactive and engaging educational content, including text, images, videos, and interactive tools, to effectively convey information about breast cancer surgery, postoperative care, and self-care strategies.
- To conduct thorough testing and quality assurance to ensure the mobile app functions correctly, is free from technical glitches, and provides a seamless user experience.
- Finalize the mobile app design and content based on feedback from patients, healthcare professionals, and other stakeholders.

### Mobile app development process

In this study, the ADDIE model was used for the development of the mobile app, as it encompasses thorough editing phases and provides repeated correction opportunities during the training material design process. Additionally, the ADDIE approach is effective in organizing health practices and patient follow-up [25, 26]. The ADDIE model is a well-established methodology for information transfer in adult education, and it has been widely utilized for the production of multimedia learning content [27]. This model is an acronym that represents the systematic implementation process for developing an educational tool [28] and it consists of the following phases: *Analysis, Design, Development, Implementation, and Evaluation.* Each phase of the model is interrelated and serves as a flexible roadmap for developing an effective training method. It’s important to note that ADDIE does not strictly follow a linear sequence of steps, and each phase is composed of different steps. Once each phase’s steps are completed, the next stage is progressed.

The table provides a comprehensive overview of how the ADDIE model is utilized in the development of mobile apps, with a thorough discussion of each stage (Table 1). The ADDIE model was employed in the creation of the mobile app due to its comprehensive editing phases and opportunities for repeated corrections during the training material design process.

**Table 1:** ADDIE stages

	Mission	Output
Analysis		
Develop learning profiles	The existing level of knowledge Symptom identification Patient characteristics	Information need Symptom statement Patient profile
Design		
Develop online learning spaces	Write objectives Develop text items Identify resources Modify technology options	Measurable objective Instructional strategy Selection avatar
Development		
Decide whether to insource or outsource tasks	Collaboration with software developers	3D video 3D video script Patient exercise movements Prototype app
Implementation		
Downloading the app to the phone	Patient training Tryout	Patient comment Data
Evaluation		
Collect and assess the data	Record data Interpret test result Revise product	Recommendation Revised prototype

### Analysis phases

The analysis phase is the initial step of the ADDIE model in instructional material design. During this stage, an overall understanding of the instructional design is established. It is a reflective stage that focuses on adopting a patient-centered approach to educational material design. In this stage, the content of the app and how it will be delivered are determined. The app’s content was generated based on responses to questions (Table 2) that were aligned with the literature and the researchers’ experiences [29–34]. These questions were developed based on current topics in the literature [13–18] for managing breast cancer symptoms. The outputs of this stage served as inputs for the design phase, and this process is further detailed in Table 2.

**Table 2:** Analysis steps

Step	Section	Output
First	Sociodemographic background	Need for information Knowledge level of breast cancer treatment Stage of breast cancer treatment
Second	Patient need	Content description Learning outcomes Content design
Third	Opinion and suggestion	Virtual presentation environment • Online • Video • Text • Animation Teaching and learning strategies
Fourth	Obstacle	Possible limitations Teaching methodology
For that reason, we asked these questions:		
Q1	What information do women undergoing breast cancer surgery need for postoperative problems?	
Q2	What are the distinctive characteristics that may affect learning?	
Q3	What are the behaviors we aim to learn?	
Q4	Which ways to transfer information?	
Q5	Which medium (video, text, animation) should be taught?	

### Design phase

In conjunction with the models, the information gathered in the analysis phase is intended to guide the acquisition of learning. In this phase, the researchers focused on the conceptual design of the mobile app, including the app environment, its functions, and how it can effectively meet the needs of patients. This phase was completed with the following steps.

#### Step 1

The content of the mobile app is designed to provide patients with information on how to prevent the development of symptoms and minimize their severity. The researchers created a 34-page text for the mobile app that covered the relevant material. An expert review was conducted to ensure that the text content was appropriate for the mobile app. The review involved analysis of the text content and scope by experts, including ten breast cancer patients who were excluded from the sample, four academic nurses, one general surgeon, and two clinical nurses. The experts agreed that certain topics, such as arm motions, lymphedema precautions, and bleeding follow-up advice, should be addressed more specifically in practice (Table 3). The researchers then edited the text content based on these recommendations, and the material to be included in the mobile app was completed.

**Table 3:** Mobile app content

Session I	Let’s manage the symptoms that may occur in the hospital
	Wound site, bleeding, and drainage, pain, infection, movement, seroma
Session II	Let’s manage the symptoms that may occur at home
	Diet, taking medications, loss of function due to limited movement (frozen shoulder), swelling in the arm (lymphedema), sleep problems, change in body image and sexual life
Session III	Let’s manage the symptoms that may occur during the survival
	Frequency of follow-up, practices to reduce the risk of recurrence, chemotherapy, radiation therapy, hormone therapy, pregnancy, nutrition, sex life, and emotional problems

#### Step 2

During this step, the researchers addressed several questions to convert the text created in the analysis phase into a mobile app. These questions included: How should the text information be presented in the mobile app? How many interfaces should be used to present the text information? How should text and video be incorporated into the pop-up module? What type of audio should be included in the video content? What visuals should be used as backgrounds for the interfaces? And what font size should be used for displaying the text to the user?

The researchers collaborated with a software engineer and a graphic designer in the production of the mobile app, which included text, photos, videos, audio, and online consultation. Based on expert recommendations, it was decided that the mobile app should have a separate interface for each symptom, with text and animation materials linked to each condition. Each symptom was presented in the appropriate period, such as in the hospital, at home, and during survival, with a different color used as the backdrop for each period. Animated videos were placed at the top of the interface, and written material was placed at the bottom. A button that allows the user to enlarge or reduce the text size was also added, and this stage of the mobile app development was completed.

### Development phase

This phase is based on both the Analysis and Design phases, and it takes the outputs of the Design phase as inputs. The mobile app was created through collaboration between the software company and the researchers. The app was produced in “Android application PacKage (apk)” format, allowing it to be downloaded onto devices. The researcher downloaded the mobile app onto the smartphones of the patients in the intervention group, taking measures to protect research data by blocking access to the platform with individual usernames and passwords for each patient, which were prepared independently in the management panel. All transactions performed using the username and password are secured with end-to-end encryption to ensure data privacy. Additionally, encryption was used to store patient messages in the online consultation section, further protecting patient privacy and personal data.

During the development of the app, the focus was on creating engaging and sustainable educational content. To ensure accessibility, content that could be appealing to multiple senses was prepared, allowing patients to listen and follow along even if they couldn’t read. Considering the patient profile, the app was designed with a three-dimensional character. The content team created ten different three-dimensional character bodies, and the most suitable character was chosen through a voting process. The animation in the app was planned to be informative, supportive, and summative. Patients and individuals fitting the patient description were allowed to vote for their preferred animation character. The character with the highest votes (56.4%) was chosen as the animated character, based on the input of 100 participants. A female voice, known for being easy to understand and listen to for long periods, was selected to convey the educational content. A theatrical performer was chosen to voice the animation, and the written information was recorded in a studio environment as clean audio without any background noise and then presented to the patients with calming background music. You can scan the QR code (quick response code) on the side to witness the character design process.

### Implementation phase

The testing phase is crucial to ensure that the mobile app functions effectively and is suitable for the target audience. A group of 5 experts, including software specialists and program developers, were enlisted to analyze various parameters. Based on their suggestions, content changes were made, such as adjusting the pop-up layout, background color flashing, and intensity of the animation sound effect in the mobile app. Additionally, feedback was sought from ten patients who had undergone surgery at a different hospital with similar characteristics as the sample. It was noted that the font size adjustment tool in the mobile app was not easily visible on the website, so it was relocated to a more prominent location on the page. Patients also suggested a larger screen size and slower narration in the video included in the app. In response, appropriate titles in the text were expanded, process stages were established, and graphics were shown in a size that could occupy the full screen with hand gestures, while the narration duration was extended. This iterative process of receiving feedback, making changes, and retesting was repeated until the app was deemed "usable". As a result, the mobile app was successfully developed to deliver its intended functionality.

### Evaluation phase

The evaluation stage assesses the effectiveness and efficiency of the process. There are two types of ADDIE assessments: formative and summative [27]. Formative assessment involves testing the impact of product usage before the final release. In the development of the mobile app, a formative evaluation process was utilized. The examination of the mobile app started from the analysis phase and progressed through each subsequent phase, with each phase undergoing its own review and evaluation process. As a result, this evaluation phase was completed by conducting evaluations at each phase.

### Prototype mobile app

**Fig. 1:**
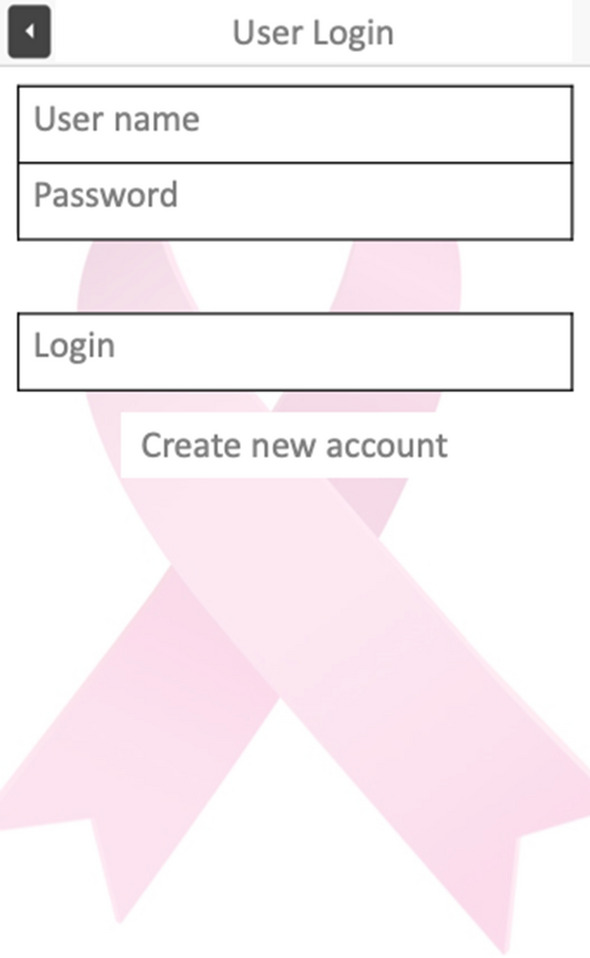
User login page

**Fig. 2:**
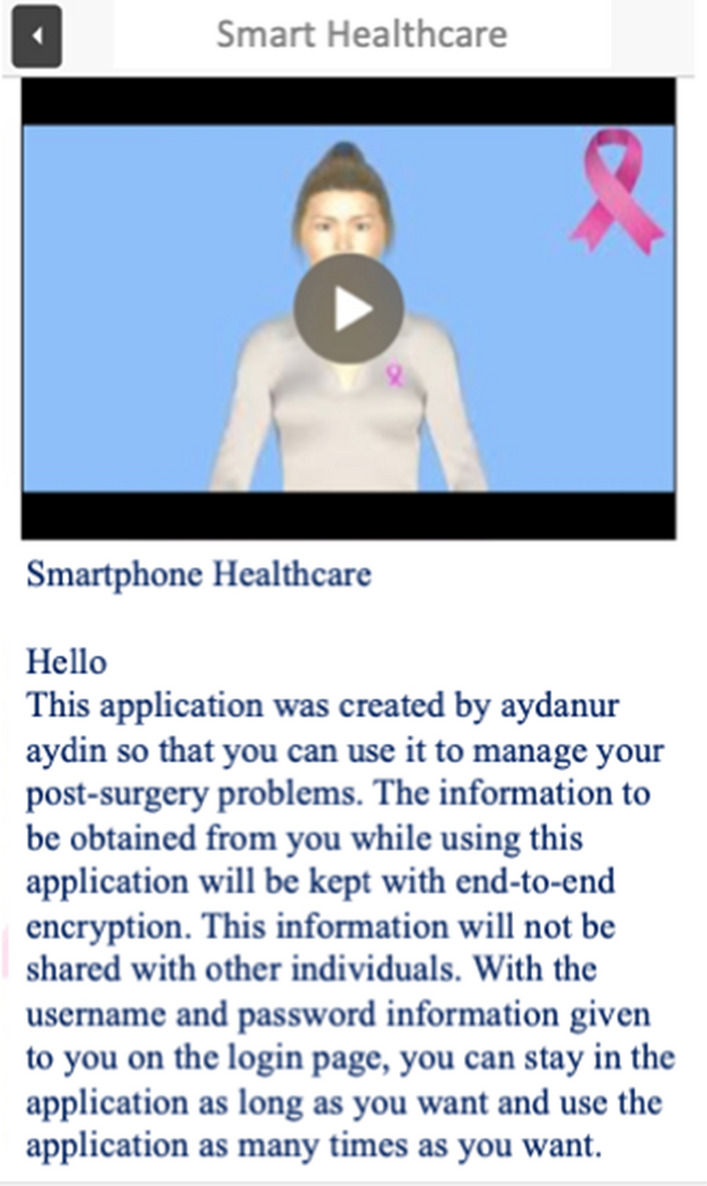
Main page

**Fig. 3:**
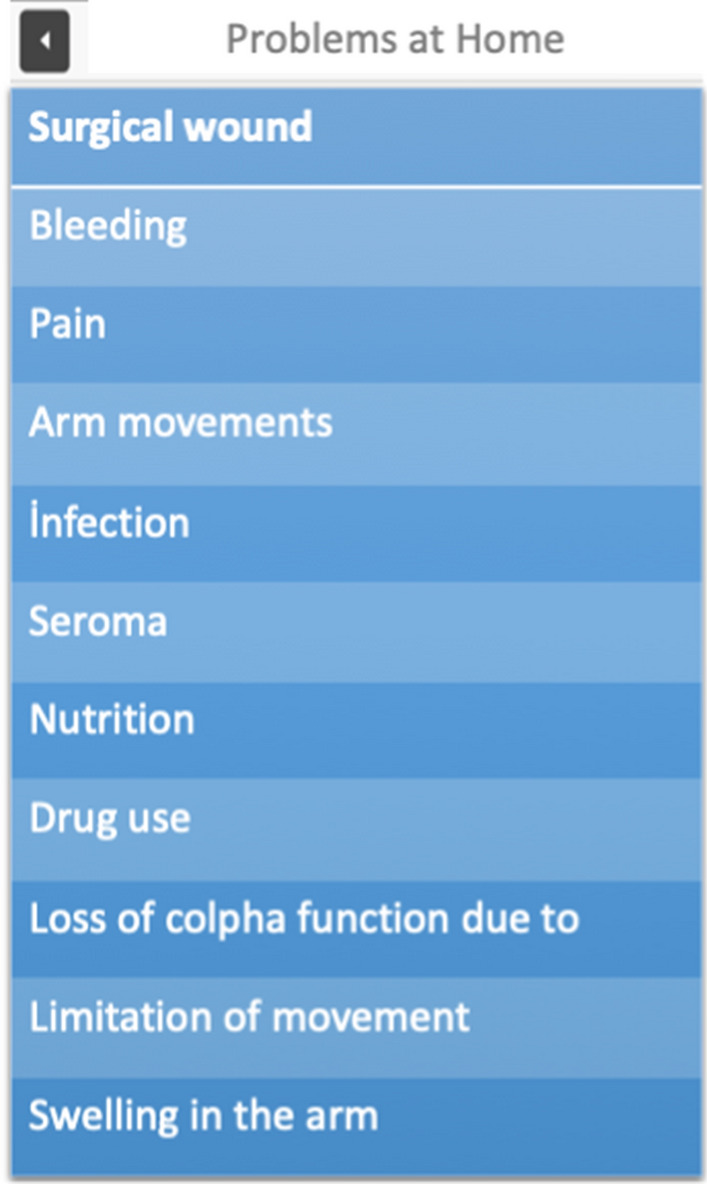
Period tab at home

**Fig. 4:**
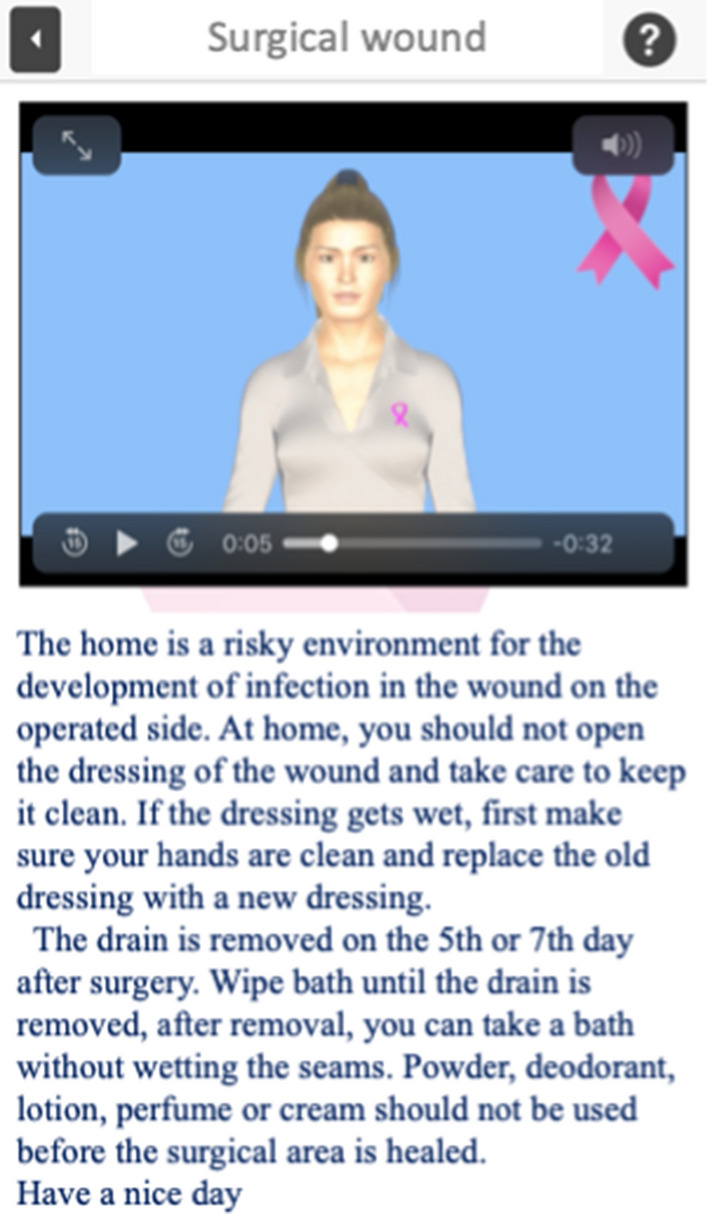
View of video and text on screen

The patient encounters the first screen as shown in Fig. 1 upon accessing the app. After inputting their information, they are directed to the main screen of the app as shown in Fig. 2. On this screen, information about the research is accessible, and the patient is asked to validate this information. The patient is then invited to select one of three options: “in the hospital”, “at home”, or “care during breast cancer treatment” based on their condition. This decision is made by the patient accordingly. After making the selection, the patient is presented with a list of issues as shown in Fig. 3. The patient is required to select the desired problem from this list. Figure 4 displays the patient's screen after making the selection. On this screen, the patient can view a video or read text information related to the selected problem. If the patient is unable to address their problem after receiving instructions on this preference, they are directed to the section indicated by an arrow. This section opens to a platform where the patient can ask questions, and those questions are delivered to the first author for prompt response (maximum 6 h).

## Conclusion

Web-based programs and mobile apps for maintaining health services are becoming increasingly popular. These programs serve valuable purposes, such as providing information, counselling, and monitoring capabilities for individuals. However, it is important to note that low-evidence apps can be downloaded from app stores and may put individuals’ health at risk. Therefore, it is essential that health professionals supervise these apps. Medical professionals should also collaborate with various disciplines, such as computer engineers, program developers, and graphic designers when creating such apps to ensure that the product serves its intended purpose effectively. To do so, healthcare workers should be familiar with computer terminology and the different forms of digital software used in app content.

The article presents a design process for mobile apps with written, verbal, and visual content that brings together different team members. These experiences can help those who are embarking on a similar path to prepare content, determine their roadmap, and navigate their way more effectively.

### Limitations

One limitation of relying on mobile apps for maintaining health services is that not everyone has access to a smartphone or reliable internet connection, which can limit the reach of these apps. Another limitation is the potential for low-quality or inaccurate health information to be disseminated through mobile apps, which can be harmful to users. Collaborating with different team members, such as computer engineers and graphic designers, can also present challenges, such as communication barriers or differences in working styles.

### Suggestions

- To address the first limitation, health professionals may need to consider alternative methods of reaching populations who may not have access to mobile apps, such as providing information through community centers or health clinics.
- To mitigate the risk of low-quality health information, health professionals could work with app developers to ensure that the content is evidence-based and reviewed by experts in the field before being disseminated to the public.
- To improve collaboration among team members with different backgrounds, it may be helpful to establish clear communication channels and establish guidelines for working together effectively. Health professionals may also consider taking courses or workshops to familiarize themselves with the terminology and software used in app development.

## Data Availability

Research data is not available.
